# Thoracentesis techniques: A literature review

**DOI:** 10.1097/MD.0000000000036850

**Published:** 2024-01-05

**Authors:** Asna Mohammed, Uri Hochfeld, Sung Hong, Davood K. Hosseini, Kevin Kim, Karan Omidvari

**Affiliations:** a Department of Internal Medicine, Hackensack University Medical Center, Hackensack, NJ, USA; b Department of Internal Medicine, Division of Pulmonary and Critical Care, Hackensack University Medical Center, Hackensack, NJ, USA; c Department of Internal Medicine, Division of Gastroenterology, Hackensack University Medical Center, Hackensack, NJ, USA.

**Keywords:** systematic review, thoracentesis

## Abstract

Thoracentesis is performed by 4 methods: gravity, manual aspiration, vacuum-bottle suction, and wall suction. This literature review investigates the safety of these techniques and determines if there is significant difference in complication rates. A comprehensive literature search revealed 6 articles studying thoracentesis techniques and their complication rates, reviewing 20,815 thoracenteses: 80 (0.4%) by gravity, 9431 (45.3%) by manual aspiration, 3498 (16.8%) by vacuum-bottle suction, 7580 (36.4%) by wall suction and 226 (1.1%) unspecified. Of the 6 studies, 2 were smaller with 100 and 140 patients respectively. Overall, there was a 4.4% complication rate including hemothoraces, pneumothoraces, re-expansion pulmonary edema (REPE), chest discomfort, bleeding at the site, pain, and vasovagal episodes. The pneumothorax and REPE rate was 2.5%. Sub-analyzed by each method, there was a 47.5% (38/80) complication rate in the gravity group, 1.2% (115/9431) in the manual aspiration group including 0.7% pneumothorax or REPE, 8% (285/3498) in the vacuum-bottle group including 3.7% pneumothorax or REPE, 4% (309/7580) in the wall suction group all of which were either pneumothorax or REPE, and 73% (166/226) in the unspecified group most of which were vasovagal episodes. Procedure duration was less in the suction groups versus gravity drainage. The 2 smaller studies indicated that in the vacuum groups, early procedure termination rate from respiratory failure was significantly higher than non-vacuum techniques. Significant complication rate from thoracentesis by any technique is low. Suction drainage was noted to have a lower procedure time. Symptom-limited thoracentesis is safe using vacuum or wall suction even with large volumes drained. Other factors such as procedure duration, quantity of fluid removed, number of needle passes, patients’ BMI, and operator technique may have more of an impact on complication rate than drainage modality. All suction modalities of drainage seem to be safe. Operator technique, attention to symptom development, amount of fluid removed, and intrapleural pressure changes may be important in predicting complication development, and therefore, may be useful in choosing which technique to employ. Specific drainage modes and their complications need to be further studied.

## 1. Introduction

Thoracentesis is a diagnostic and/or therapeutic procedure performed by aspiration of fluid from the pleural space. The procedure is performed by 1 of 4 methods: manual suction via syringe, gravity drainage, using continuous suction from a vacuum bottle, or using continuous suction through a wall system. There are currently no clear guidelines or standard of care on which method to use and it is largely preference based with institutional variations and limitations. While a relatively low-risk procedure, major complications include pneumothorax, re-expansion pulmonary edema (REPE) and bleeding that can lead to increased morbidity, mortality, and healthcare costs.^[1,2]^ Patients can also experience minor complications including symptoms of chest discomfort, shortness of breath and cough during or after the procedure. There are various proposed factors associated with complications including, underlying etiology, history of bleeding disorders, coagulopathy, the method used, the amount and the speed of fluid removal, and the negative pressure created during the procedure. There have only been few studies that explore the relation between complications of thoracentesis with the mode of fluid removal. We aim to review whether the mode of drainage during thoracentesis affects the rate of complications.

### 1.1. Study design

Literature review.

Hackensack Meridian Health Institutional Review Board approval was not necessary because this is a literature review study.

## 2. Result

This is a literature review of 6 studies that included 20,815 thoracenteses, 80 (0.4%) of them were performed by gravity drainage, 9431 (45.3%) by manual aspiration, 3498 (16.8%) by vacuum, 7580 (36.4%) by wall suction and 226 (1.1%) were unspecified (Table 1). Overall, there was a 4.4% complication rate. The majority were minor complications such as chest discomfort, bleeding at the site, and vasovagal episode. The major complications included REPE, pneumothorax, and hemothorax. The rate of pneumothorax and REPE was 2.5%. Sub-analyzed by each group, there was a 1.2% complication rate in the manual aspiration group, (0.7% pneumothorax or REPE), 8% in the vacuum group (3.7% pneumothorax or REPE, 4.3% minor complications), 4% in the wall suction group (all of which were either pneumothorax or REPE), and 73% in the unspecified group (most of which included vasovagal episodes).

**Table 1:** Summary of thoracentesis complications.

Mode of drainage	Thoracentesis	Any complication	PTX	REPE	Others*	Chest discomfort/pain at site/cough
Total	20,815	913	499	19	173	222
Gravity	80 (0.4)	38	0	0	0	38
Manual hand suction	9431 (45.3)	115	58	10	17	30
Vacuum	3498 (16.8)	285	126	2	3	154
Wall	7580 (36.4)	309	302	7	0	0
Unspecified	226 (1.1)	166	13	0	153	0

(%);

* Others = hemothorax, bleeding at site, vasovagal episode, hypotension.

PTX = pneumothorax, REPE = re-expansion pulmonary edema.

In the largest study to date, Sagar et al performed a retrospective cohort study examining the incidence of complications (including pneumothorax, REPE) of 10,344 thoracenteses performed by symptom-limited suction drainage either by vacuum bottle (27%) or wall suction (73%) over a 14 years span. The incidence of pneumothorax was 3.98% (0.28% required intervention), REPE 0.08% and mortality from thoracentesis was 0.03%. In the vacuum group, there were 110 cases of pneumothorax and 1 case of REPE; in the wall suction group, there were 302 pneumothoraces and 7 REPE. In addition, there were 150 other complications reported, including hemothorax, bleeding and mostly vasovagal episodes. The sample study was large and demonstrated symptom-limited suction drainage of pleural fluid is safe with a low risk of complications even in those with large volumes drained.^[3]^

Lentz et al performed a prospective, multicenter, single-blind, randomized controlled trial (GRAVITAS study) that investigated whether active aspiration (i.e., manual aspiration using a syringe) or gravity drainage during thoracentesis affected complication rates including chest discomfort, REPE, and pneumothorax. The study included 140 patients and showed that there were no differences in procedural chest comfort (*P* = .17), post-procedure (24, 48 hours) chest discomfort or breathlessness (*P* = .85, 0.77, resp.) with only 1 pneumothorax reported in the suction group, and no cases of REPE. However, the primary difference between the 2 groups was the procedural duration which was less in the suction group (mean difference 7.4 minutes; *P* < .001). Overall, this study demonstrated that there were no significant differences between active pleural fluid aspiration and gravity drainage in relation to patient discomfort or other significant complications and that they are both comparable in safety.^[4]^

Senitko et al performed a single-center randomized study comparing the safety, pain level, and time involved in vacuum (n = 51) vs manual (n = 49) thoracenteses. They noted that vacuum thoracenteses led to a statistically significant increase in all cause complications (5 vs 0, *P* = .03), pneumothorax (3 vs 0), hemothorax that led to death (1 vs 0), REPE causing respiratory failure (1 vs 0). Early termination was also more prevalent in vacuum vs manual drainage (8 vs 1, *P* = .018) as well as statistically significant higher reports of pain (*P* < .05). The authors suggested that these reported findings may be related to higher pressure and shorter duration seen in vacuum versus manual suction.^[5]^

Ault et al performed a prospective cohort study of 9320 thoracenteses to evaluate specific demographic and clinical factors that have been commonly associated with complications of thoracentesis such as pneumothorax, REPE and bleeding. The procedure was performed by manual hand aspiration. There were 0.61% iatrogenic pneumothoraces, 0.01% REPE, and 0.18% bleeding episodes. The overall complication rate (0.98%) for this large group of patients was overall low.^[6]^

Elyah and Chatterji published a prospective study of 658 ultrasound assisted thoracenteses performed using vacuum bottle drainage. They analyzed the incidence of symptoms and complications, and possible risk factors associated with symptoms development. Of the 658 thoracenteses, 24% experienced minor complications including cough in 56.4% and pain (either chest or throat) in 52%. Large volume fluid removal was associated with a higher risk of developing symptoms or major complications (*P* = .002) including pneumothorax (*P* = .01). Furthermore, they noted that thoracic ultrasound estimations of pleural effusion volume pre-procedure, and the quantity of volume removed, were both predictive of pain (*P* = .001). The authors concluded that the volume of aspirate, rather than technique, seems to be associated with symptoms development.^[7]^

Petersen and Zimmerman published a study in 2000, reviewing 278 thoracenteses to assess the utility of routine chest radiography following thoracentesis. They demonstrated that in the absence of a complication during the procedure, routine chest radiography following a thoracentesis is not indicated. Utilization of vacuum bottles was noted to be a risk factor associated with pneumothorax development (OR, 4.6; *P* < .01), postulating that perhaps a vacuum device limits detection of free air aspiration. It was also noted that a higher percentage of pneumothoraces that developed with vacuum bottle utilization required a subsequent chest tube, suggesting a potential increased severity of complication with vacuum bottle usage in thoracenteses.^[8]^

## 3. Discussion

This literature review aims to establish whether there is a difference in complication rates between thoracenteses performed via gravity versus different suctions techniques. Only 6 articles were identified in the literature that addressed this question. There was a 4.4% complication rate out of 20,815 thoracenteses including pneumothorax, REPE, chest discomfort, bleeding at the site, vasovagal episode, and hemothorax. The rate of major complications including pneumothorax and REPE alone was 2.5% from any mode of thoracentesis. The manual aspiration group had a 0.7% risk of pneumothorax or REPE, the vacuum group 3.7% and 4% in the wall suction group.^[3–8]^ Overall, the complication rates were low and there are no significant studies that have determined that any method of drainage has a direct impact on development of complications from thoracentesis. The cause of these complications is multifactorial and technique has not been proven to be a major factor.

The studies performed by Sagar et al and Lenz et al (GRAVITAS) were comparatively larger studies that specifically addressed the comparison of the various drainage modalities. The GRAVITAS study demonstrated that procedural duration was less in the suction group compared to gravity drainage. Otherwise, there were no significant differences between active pleural fluid aspiration and gravity drainage in relation to patient discomfort or other significant complications, suggesting that both techniques are comparable in safety.^[4]^ Moreover, the study by Sagar et al was the largest study and while they only included patients who underwent suction drainage by either vacuum or wall suction, their overall rate of major complications remained low (3.98% pneumothoraces with only 0.28% requiring intervention).^[3]^

The shorter duration of thoracentesis via suction was noted by Senitko et al, who also reported that vacuum thoracenteses led to a statistically significant increase in complications including pneumothorax, hemothorax, REPE causing respiratory failure, and early termination of the procedure as well as statistically significant higher reports of pain.^[5]^ However, this study had a very small number of patients and the statistical significance was only reached when all disparate complications were clumped into one. Similarly, Petersen and Zimmerman also noted higher incidence of pneumothoraces in thoracenteses with suction devices.^[8]^ Elyah et al noted that cough and pain (chest or throat) were present in more than 50% of cases drained by vacuum, but it was particularly associated with large volume removal.^[7]^ While suction drainage was noted to have slightly higher complication rates, the respective studies had limitations. For example, in one study chest radiography was not routinely ordered after the procedure, so asymptomatic cases of pneumothorax were likely missed.^[8]^ Other studies had smaller sample sizes and potentially could have included cases of unexpandable lung.^[5,6,8]^ A proposed mechanism for reduced tolerability to the procedure via suction includes faster pressure shift in the pleural space.

There are likely other factors that contribute to the rate of complications during thoracentesis independent of the method of drainage including procedure duration, quantity of fluid removed, and operator technique. For example, pneumothorax, the most commonly described major complication from thoracentesis, has been associated with increased number of needle passes through the skin, drainage volume greater than 1.5 L, and underweight BMI.^[9]^ Ault et al found that underweight patients were 3 times more likely to develop pneumothorax which could be attributed to the smaller distance between the lung and chest wall.^[7]^ Furthermore, studies have shown REPE to occur with large amounts of fluid removal and so reviews have suggested to limit drainage to 1 to 1.5 L. However, since then, other studies have suggested that this risk is more likely related to intrapleural pressure and recommend symptom limited drainage instead.^[3,10]^ For instance, Sagar et al showed symptom-limited suction drainage of pleural fluid using vacuum or wall suction is safe with a low risk of complications even in those with large volumes drained.^[3]^ Additionally, emphasis on operator experience and the use of ultrasound have had a positive impact in reducing the rates of complications.^[9]^ For example, the use of ultrasound has played a big role in the reduction of complications, particularly pneumothoraces, with one observational study reporting up to 19% reduction in development of pneumothorax with the use of ultrasound.^[2]^

## 4. Conclusion

The overall rate of significant complications from thoracentesis by any technique remains low. All suction modalities of drainage remain safe and operator technique, attention to symptom development, amount of fluid removed, and intrapleural pressure changes may play a larger role in thoracentesis complication development. Further randomized trials are needed to study how the specific modes of drainage during thoracentesis affect the development of these complications.

## Author contributions

**Conceptualization:** Karan Omidvari

**Data curation:** Asna Mohammed, Uri Hochfeld, Sung Hong, Kevin Kim.

**Formal analysis:** Karan Omidvari.

**Investigation:** Karan Omidvari.

**Methodology:** Karan Omidvari.

**Supervision:** Davood K. Hosseini.

**Writing –original draft:** Karan Omidvari.

**Writing –review & editing:** Davood K. Hosseini, Karan Omidvari.
